# Tuning PVDF Membrane Porosity and Wettability Resistance via Varying Substrate Morphology for the Desalination of Highly Saline Water

**DOI:** 10.3390/membranes13040395

**Published:** 2023-03-30

**Authors:** Turki N. Baroud

**Affiliations:** 1 Materials Science & Engineering Department, King Fahd University of Petroleum and Minerals, Dhahran 31261, Saudi Arabia; turkibaroud@kfupm.edu.sa; 2 Interdisciplinary Research Center for Membranes & Water Security, King Fahd University of Petroleum and Minerals, Dhahran 31261, Saudi Arabia

**Keywords:** wettability, membrane distillation, polymeric membrane, phase inversion, substrate

## Abstract

Here, we report the fabrication of a series of highly efficient polyvinylidene fluoride (PVDF) membranes via substrate morphology variations. A wide range of sandpaper grit sizes (150–1200) were utilized as casting substrates. The effect of the penetration of abrasive particles present on the sandpapers on the casted polymer solution was tuned, and the impact of these particles on porosity, surface wettability, liquid entry pressure and morphology were investigated. The membrane distillation performance of the developed membrane on sandpapers was evaluated for the desalination of highly saline water (70,000 ppm). Interestingly, the utilization of cheap and widely available sandpapers as a substrate for casting can not only help in tuning the MD performance, but also in producing highly efficient membranes with stable salt rejection (up to 100%) and a 210% increase in the permeate flux over 24 h. The findings in this study will help in delineating the role of substrate nature in controlling the produced membrane characteristics and performance.

## 1. Introduction

The pretreatment of produced water (removal of pollutants and contaminants, removal of heavy metals, separation of dissolved solids, etc.) results in brine with a salt concentration that depends on the native geology of the relevant well [1]. While the brine resulting from the treatment of produced water is better suited to re-injection for other fracking jobs, it can also be used for other purposes (irrigation, industrial water, etc.) [2,3,4]. However, the utilization of the treated produced water for these purposes requires additional steps to reduce the water salinity [5,6,7]. Thus, desalination techniques can be employed to achieve the ultimate goal. Membrane distillation (MD) is a promising emerging technology for water desalination [8,9,10,11]. Interestingly, MD not only offers a solution to overcome the high salinity of the treated produced water directly, but can also be integrated with an RO unit to treat and reuse the highly saline RO concentrate which would otherwise be discarded [12]. Notably, the relatively low working temperature and the low sensitivity towards the feed-water salinity (as the water vapor is less impacted by the feed salinity compared to RO) make the MD process an attractive technique for producing high-quality distillate [13,14,15]. The membrane is the heart of MD technology. Membrane hydrophobicity is a crucial characteristic for highly efficient MD membranes, as only water vapor is allowed to penetrate from one side (hot stream) to the other (cold stream). Typically, hydrophobic surfaces have contact angles larger than 90 °C according to the Young equation [16]. The large pore sizes, high hydrophobicity and low surface energy of MD membranes make them suitable for desalinating the pre-treated produced water. Due to the limitations associated with commercial membranes, the development of membranes with superior performance (with highly hydrophobic surfaces, high wetting resistance, high permeability, controlled porosity, etc.) is considered a cornerstone for the further advancement of MD for water desalination [8,17,18,19,20,21,22].

To date, several hydrophobic polymeric membranes have been utilized for membrane distillation, including polyvinylidene fluoride [23,24,25,26,27], polytetrafluoroethylene [28,29,30] and polypropylene [31,32,33,34]. Polyvinylidene fluoride polymer with low surface energy has gained much interest in membrane distillation due to its mechanical and thermal properties, as well as the ease of processability of polyvinylidene fluoride which allows better control over the porosity, pore distribution and functionalization of the membrane. Phase inversion is one of the commonly used methods to convert the polymeric dope solution into a continuous solid porous membrane. In non-solvent-induced phase inversion, the casted polymer-doped solution is immersed in a coagulation bath where the solvent–nonsolvent exchange takes place instantaneously. The solvent–nonsolvent exchange results in a solid phase which will form the membrane structure and a liquid phase which will ultimately form the porosity of the membrane [35,36]. This phase inversion will result in the formation of an integral asymmetric structure composed of a highly dense skin layer and a more porous layer with different possible cavities and structures (i.e., finger-type, sponge-type) [36,37,38]. The nature of the skin layer and the underlying porous layer depends on many factors, including the nature of the polymer used, the solvent, the non-solvent, the coagulation bath temperature and the substrate’s chemistry and roughness.

During the formation of the polymeric membrane, the casted solution undergoes significant shrinking, and considerable shrinking stress is built up during the precipitation of the polymer solution. The nature of the cavities (type, propagation, size) in the structure of the polymeric membrane will be significantly affected by this shrinkage [39]. In many separation processes, control of the porosity (size, amount, shape) is simultaneously crucial for high selectivity and high flux. Thus, several reported approaches considered enhancing the membrane performance by controlling the polymer solution, phase inversion parameters and post-treatment [36,40]. Higher water contact angles for chemically modified surfaces were reported in [41,42,43]. However, chemical modifications (alone) of a surface normally introduce limited/unstable enhancement of the contact angles. Interestingly, highly hydrophobic (superhydrophobic) surfaces with a high contact angle (≥150°) can be achieved by controlling and tuning the surface roughness [44,45]. Inorganic fouling (scaling) and membrane wetting are considered among the main challenges that limit the long-term stability and salt rejection of the membrane distillation technology [46,47,48,49]. Besides other approaches that can mitigate the wetting and fouling problems, controlling the membrane surface characteristics has been considered an essential approach toward tackling and reducing the scaling propensity [47,50]. More specifically, increasing the surface roughness and lowering the surface energy are strategic approaches toward fabricating excellent membrane candidates for membrane distillation technology [43]. Several studies have reported the successful improvement of surface hydrophobicity by controlling the membrane roughness [44,51,52,53,54,55]. Controlling the additives and the deposition of nanoparticles on the membrane surface are among the typically reported approaches for increasing the membrane roughness [48,56,57,58,59]. However, most of the reported approaches to creating roughness are time-consuming and involve complicated steps, and might not be scalable.

Thus, it is important to have a facile and scalable approach to introduce membrane surface roughness that can lead to a Cassie–Baxter metastable state, which allows gas pockets to be trapped between the surface and the liquid (solid–gas interface) and result in a superhydrophobic surface and high water repellency. Recent studies explored the impact of substrate nature (i.e., surface energy, chemistry, roughness) on the formation of membranes and their characteristics [26,60,61]. Such surface engineering can assure the fabrication of membranes that demonstrate excellent anti-fouling characteristics and high wetting resistance. However, enhancing the membrane porosity and wettability resistance via substrate variation to enhance the vapor diffusion across the membrane for membrane distillation of highly saline water is rarely investigated.

In this work, we aim to maximize the value of the waste stream that is normally faced in the oil and gas industry. This study helps to push the limit of membrane performance via a facile and scalable membrane fabrication approach for the membrane distillation of highly saline water (70 g/L). The reported approach was adopted to fabricate a wide range of microporous PVDF membranes with endowed surface roughness and enlarged porosity via the utilization of different sandpaper substrates for the desalination of highly saline water. The reported results provide interesting insights into the possibility of simultaneously enhancing the membrane rejection and flux without primarily relying on additives and sacrificial materials. Additionally, the findings can help in better matching the right substrate morphology with the needed membrane characteristics. Thus, the findings can pave the way for microporous polymeric membranes in wide industrial applications.

## 2. Experimental

### 2.1. Materials

N,N-Dimethylformamide (C3H7NO; 99%), polyvinylpyrolidone powder (C6H9NO)n (average Mw ~10,000) and poly (vinlylidene fluoride) powder (CH2CF2)n (average Mw ~534,000) were purchased from Sigma Aldrich (St. Louis, MO, USA). In this work, deionized water from the Millipore water purification system was used for the membrane fabrication.

### 2.2. Membrane Fabrication

The polymer dope solution was prepared by dissolving PVDF polymer in N,N-dimethylformamide (DMF) solvent with the ratio of 15:80. The mixture was stirred for 24 h before the addition of 5% polyvinylpyrrolidone (PVP) as a pore former in the membrane matrix. The final solution was stirred to attain a homogeneous solution. Then, the polymer dope solution was casted on a glass and a wide range of textured substrates with a casting thickness of 250 μm. Following this, the casted solution was immediately immersed in a coagulation bath containing DI water (80 vol%) and 2-propanol (20 vol%) with the ratio of 80:20 at a controlled temperature of 30 °C for 10 min. The precipitated membranes after demixing were transferred to another bath with only DI water for 24 h. Finally, the membrane was left to dry at room temperature for 24 h. In the following, PVDF-G refers to the PVDF membrane casted on a glass substrate while PVDF-SX stands for the casted PVDF membrane on sandpaper with different grit numbers X that represent the roughness of the sandpaper surface. In this work, a wide range of sandpapers (waterproof and electro-coated silicon carbide abrasive paper, Korea) with different grit numbers X (S150, S220, S400, S600, S1200) were used as a substrate for the casting of the polymer solution. A smaller grit number indicates a rougher sandpaper surface, while a larger grit number indicates a smoother surface with fine grit. For example, PVDF-S150 refers to a membrane that was cast on sandpaper with a grit number of 150.

### 2.3. Membrane Characterization

A field-emission scanning electron microscope FE-SEM (FEI Quanta 250 FEG, USA) was used to characterize the top and cross-section surface morphology of the fabricated membranes. To characterize the membrane cross section, the samples were frozen in liquid nitrogen before they were fractured. A sputter coater was used to coat all the samples with a thin layer of platinum. Optical images for the top surface of the sandpaper were taken by Olympus dsx510. To evaluate the wettability of the membrane surface, a goniometer instrument (DM-501, Kyowa Interface Science Co. Ltd., Niiza, Japan) was used to measure the water contact angle. The sessile drops were placed on the membrane in five different spots to obtain the average value of the contact angle. To measure the minimum transmembrane pressure needed for the water droplet to penetrate across the membrane, liquid entry pressure (LEP) was measured via a custom-built instrument. The pressure was slowly increased by pressurizing a small chamber of water against the membrane. The pressure at which the initial drop of water was noticed on the membrane surface was recorded. For reproducibility, this process was repeated at least three times and the average LEP value was reported. The porosity of the glass substrate and textured membranes were determined using the gravimetric method and the resulting values were calculated from Equation (1). The membrane samples were weighed and recorded as the weight of dry membranes. Then, the samples were fully soaked in isopropanol until it filled the membrane pores, and samples were weighed and recorded as the weight of the wet membrane.

(1) $$\varepsilon \%=\frac{\left(m_{w}-m_{d}\right)/d_{i}}{\left[\left(m_{w}-m_{d}\right)/d_{i}\right]+(m_{d}/d_{p})}$$

where ε is the membrane porosity, m_w_ is the weight of the wet membrane and m_d_ is the weight of the dry membrane. d_i_ is the density of isopropanol (0.785 g/cm^3^), and d_p_ is the density of the PVDF membranes (1.74 g/cm^3^). The pore size and pore size distribution were measured using the porometer equipment. The membrane samples were soaked with profile fluid and fixed into the chamber, which was pressurized within a pore size range. The pore sizes were recorded, and the pore distribution was plotted, representing the individual membrane. The thickness of the membranes was recorded in a different area of the membrane using Mitutoyo VL-50A LITEMATIC digital measuring instrument.

### 2.4. Membrane Performance

**Figure 1:**
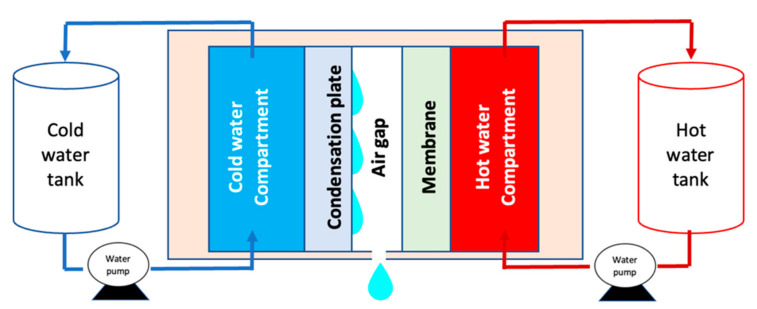
Schematic diagram of the air-gap setup used for the desalination of highly saline water.

Lab-scale air-gap membrane distillation (shown in Figure 1) was used to examine the performance of the fabricated membranes on glass and textured surfaces. All developed membranes were fixed into a stainless-steel membrane module. Highly saline water of 70 g/L NaCl was used as the feed solution. The feed was heated to 70 °C and channeled through the cell continuously. In the setup, vapor is generated and condenses to liquid in the coolant side. The permeate is collected and measured using an electrical balance and the values are recorded. The salt rejection is also measured from the conductivity meter. The experiment was run for 24 h, and six types of membranes were tested, representing the casting substrate on glass and various grades of sandpaper for textured membranes. Table 1 illustrates the testing conditions for all membranes.

The permeate flux of the membrane is calculated from Equation (2):

(2) $$\mathrm{Permeate}\;\mathrm{Flux}\;J=\frac{V_{p}}{A_{E}t}\;$$

where $V_{p}$ is the volume of collected permeate in L, $A_{E}$ is the effective area in m^2^, t is the time taken for permeate collection, estimated in hours, and J is the permeate flux in L/m^2^·h.

The salt rejection S.R is calculated using Equation (3):

(3) $$\mathrm{Rejection}\;\mathrm{Factor}\;S.\;R\%\;=\left(\frac{C_{f}-C_{p}}{C_{f}}\right)\times 100\%$$

where R, $C_{f}$ and $C_{p}$ are the rejection (%), the concentration of feed in ppm and the concentration of permeate in ppm, respectively.

## 3. Results and Discussion

**Figure 2:**
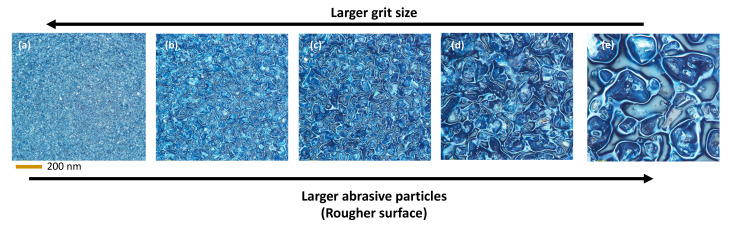
Top-surface optical images of sandpapers with different grit sizes: (**a**) S1200, (**b**) S600, (**c**) S440, (**d**) S220 and (**e**) S150.

Sandpaper is typically used to smooth surfaces, and can be classified according to the size of the abrasive particles glued onto the sandpaper. Thus, sandpaper can be differentiated according to the grit number, which is inversely correlated to the abrasive particle size [62]. A larger grit size indicates a finer surface, while a smaller grit number indicates a rougher surface with larger abrasive particles. In this study, several sandpapers with different grit sizes were used, from smoother to rougher sandpaper surfaces (S1200 to S150) [63,64]. Figure 2 shows the optical images of different sandpapers used in this study as substrates for casting. As the grit number decreases from S1200 to S150, the abrasive particle size increases accordingly. Additionally, even within each sandpaper, the silicon carbide particles on the sandpaper vary in terms of size and shape. Notably, the density of the abrasive particles (number of particles/unit area) on the sandpaper is higher for higher grit numbers. This can be ascribed to the way finer particles are packed per unit area. The possibility of creating roughness on the membrane surface by casting on sandpaper and then peeling off is explored in this study. A smooth surface with finer particles might not noticeably create roughness on the membrane surface after the formation of the membrane during the phase inversion. Conversely, casting on rougher surfaces (i.e., S150) allows noticeable penetrations on the casting solution, and accordingly influences the membrane surface morphology and textural characteristics.

**Figure 3:**
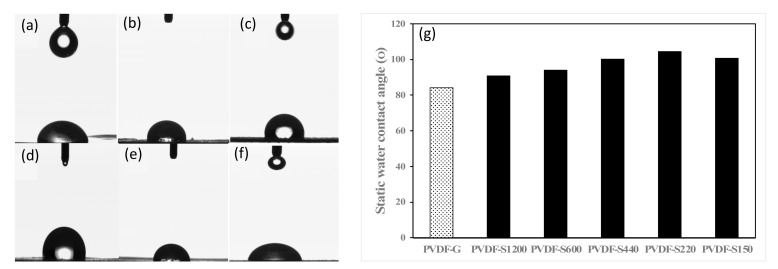
Water droplet images on (**a**) PVDF-G, (**b**) PVDF-S150, (**c**) PVDF-220, (**d**) PVDF-S440, (**e**) PVDF-S600 and (**f**) PVDF-S1200 surface. (**g**) Comparative wetting behavior of PVDF-G and PVDF-SX series.

In membrane distillation, contact angle (CA) is typically used to measure the hydrophobicity of the microporous membrane by determining the ability of a solid to repel a liquid. Thus, a higher contact angle leads to higher resistance to the wettability of the substrate [65,66,67]. When a water droplet on a substrate has a CA larger than 90, this surface is classified as hydrophobic (i.e., water-repellent); a surface with CA larger than 150 is considered to be superhydrophobic [68,69]. Figure 3 depicts the CA values for the developed membranes where casting in glass PVDF-G resulted in a hydrophilic membrane with CA around 84. Notably, casting on sandpapers PVDF-SX resulted in hydrophobic membranes ranging from around 91 to 105 degrees. Although all the membranes were fabricated by the same polymer, solvent, sandpaper materials and fabrication conditions, variations in the roughness of the substrate can enhance the hydrophobicity of the membranes [60]. The increase in water contact angle for the PVDF-SX series can be attributed to the printed roughness on the membrane surface as a result of casting on rough surfaces. Notably, the increase in CA levels is around 100 degrees. The endowed roughness on the membrane surface of the PVDF-SX series helped to increase the number of air pockets trapped on the membrane surface. A similar enhancement of water contact angle was previously reported as a result of casting on rough surfaces. The advantage of the reported approach on casting on sandpaper substrate is that it offers an easier way of peeling off compared to mesh substrates [26] and other surface-roughening substrates [60].

**Figure 4:**
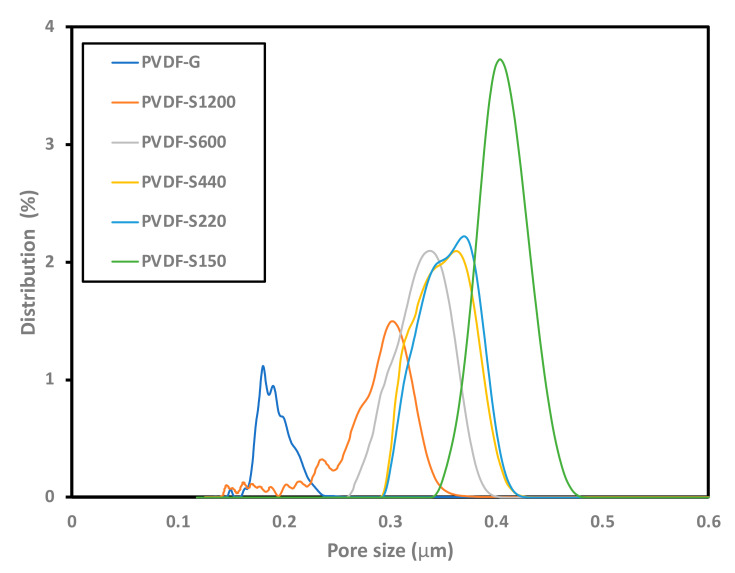
Pore size distribution of PVDF-G and PVDF-S series membranes.

Liquid entry pressure (LEP) is a critical characteristic of hydrophobic membranes that is used in membrane distillation applications. LEP is the transmembrane minimum pressure needed to allow water droplets to penetrate across the membrane through the largest pore [70]. Thus, a higher value of LEP indicates a higher wetting resistance. LEP depends on the maximum pore size and the hydrophobicity of the membrane surface (measured by the contact angle). Therefore, the hydrostatic pressure on the feed side must be maintained below the LEP value to avoid penetration of the solution across the membrane to the permeate side. Therefore, although increasing the pore size can lower the mass-transport resistance and help in increasing the permeate flux, it might help in minimizing the value of LEP and accordingly result in wetting the membrane [71]. Thus, it is necessary to optimize the increase in the pore size and the contact angle to result in a high LEP [72]. Table 2 presents the porosity percentages of all the developed membranes. The porosity was enhanced from 73% to 81% when a highly rough substrate (S150 grit) was used instead of casting smooth glass substrate. Interestingly, not only could the porosity percentage be enhanced, but also the range of pore sizes in the membrane matrix. Table 2 and Figure 4 show the range of pore size and pore size distribution, respectively, for all the developed membranes on different substrates. Casting on a glass substrate resulted in a maximum pore size of around 0.21 μm, a mean pore size of around 0.18 and a minimum pore size of around 0.16 μm. Notably, casting on all the sandpapers with different extents of roughness shifted the range of pore size towards higher values. This was obvious even when a substrate with minor roughness (1200 grit size) was used. Interestingly, casting on 150-grit sandpaper (which is the roughest substrate in this work) to make PVDF-S150 resulted in almost double the pore size range in the membrane. Table 2 presents the LEP values for the fabricated membranes. Interestingly, casting on a rough surface did not necessarily result in enhanced LEP values. Since sandpapers with different grit numbers were used as casting substrates, the textured surface varied in terms of contact angle; thus, increasing the pore size did not necessarily result in decreasing the LEP, as the LEP is influenced by both values (max pore size and contact angle). The penetration of sandpaper abrasive particles on the casted solution can simultaneously increase the membrane roughness and enhance the membrane porosity by limiting the shrinkage of the membrane during the phase-inversion step. Thus, a substrate with roughness can increase the maximum pore size, and if this was not combined with an increase in the contact angle then this rough surface would inversely lower the LEP. Interestingly, casting on 400 grit number resulted in PVDF-S400 with a membrane that possessed an optimum combination of CA and maximum pore size, and accordingly resulted in the highest value for LEP (1 bar).

**Figure 5:**
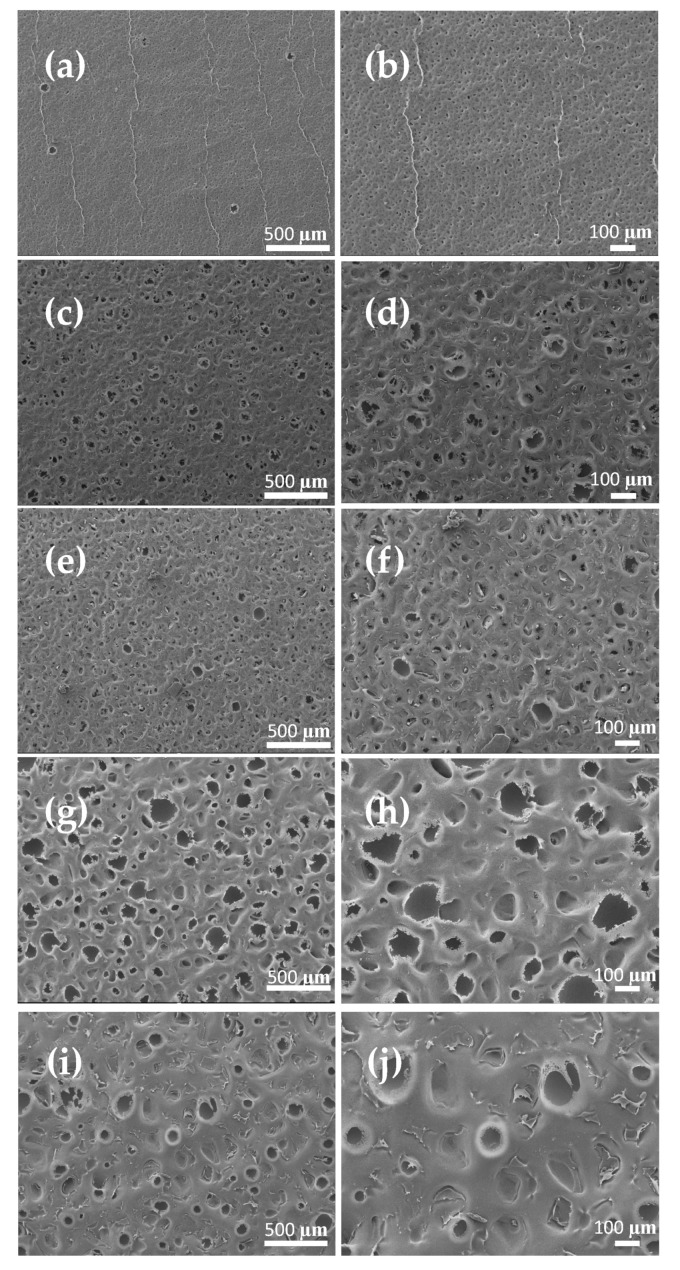
Top-surface FE-SEM images of PVDF-S series membrane at different magnifications: (**a**,**b**) PVDF-S1200, (**c**,**d**) PVDF-S600, (**e**,**f**) PVDF-S440, (**g**,**h**) PVDF-S220, (**i**,**j**) PVDF-S150.

Figure 5 shows the morphology of the membrane surface. Notably, casting on all sandpapers resulted in fragmented holes randomly distributed through the membrane surface. The size of the holes and their quantity were positively correlated with the size and number of particles on the sandpaper. For instance, PVDF-S1200 had a small number of small holes compared with PVDF-S150. These basins (holes) represent the negative replica of the sandpaper surface (e.g., Figure 5h,j). Notably, the sandpaper intrusions in the casted dope solution were able to influence the amount of shrinkage the membrane could experience following the phase inversion and drying step (see Table 2). Considering the initial casting dimension, casting on a smooth glass surface resulted in around 8% shrinkage compared to the final membrane dimension. However, casting on relatively more rough surfaces could slightly decrease the shrinkage %, as can be seen in PVDF-S1200. Shrinkage of the final membrane dimension could be completely eliminated by casting on a rougher surface (i.e., S600, S440, S220, S150). Hence, casting on sandpaper could potentially enhance the flux of the membrane, as the extent of shrinkage and contraction of the membrane (and its porosity) would be avoided or at least minimized [39,73].

**Figure 6:**
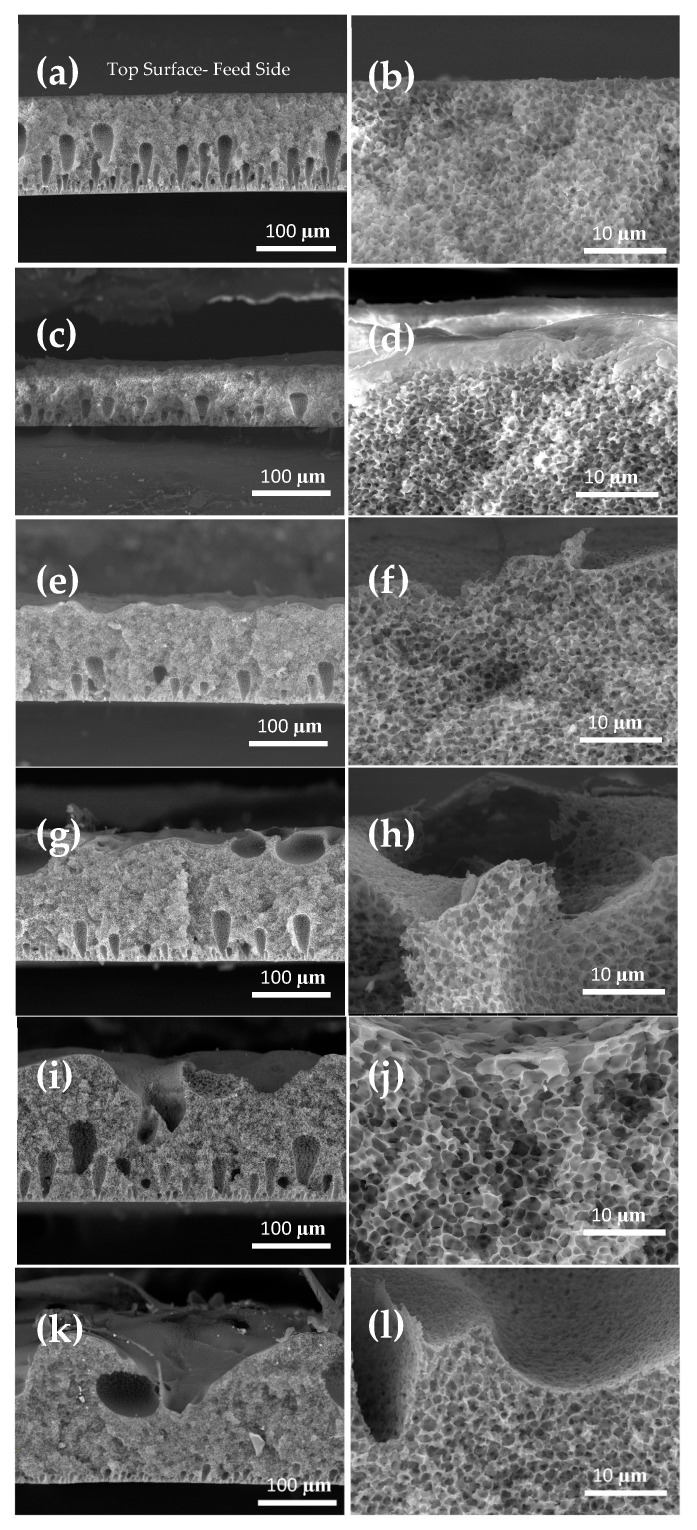
Cross-sectional FE-SEM images of (**a**,**b**) PVDF-G, (**c**,**d**) PVDF-S1200, (**e**,**f**) PVDF-S600, (**g**,**h**) PVDF-S440, (**i**,**j**) PVDF-S220 and (**k**,**l**) PVDF-S150 at different magnifications. Top side is the hot water feed side.

Figure 6 illustrates the cross sections of the developed membranes by casting the dope solution in different substrates. There are three elements of the structure (morphology of the top surface, finger-like channels, porosity) that exist in all membranes, but they differ in their nature. Clearly, the substrate nature not only influences the morphology, as shown in Figure 5, but can also affect the morphology and structure of the membrane. Casting on smooth surfaces such as a glass substrate (e.g., PVDF-G) led to a dense layer and finger-like structure with more depth of the finger-like channels [74]. Conversely, the casted membrane on a rougher surface (i.e., PVDF-S150) showed minimal penetration of the fingers throughout the cross section of the membrane. The investigated range of grit-size sandpaper helped in understanding the role of surface roughness and possible strategies to control the membrane structure. Finger-like morphology was gradually minimized going from smooth substrates such as glass and S1200 grit size to more rough surfaces such as S220–S150 sandpapers. This can be attributed to the fact that during the immersion of casted solution in the coagulation bath containing the participant (non-solvent) the immediate formation of the skin layer occurred via the precipitation of polymer-rich phase from the initial homogeneous solution. This skin layer acted as a barrier between the non-solvent and the rest of the casting solution. The finger-like structure went through initiation and propagation stages. The initiation of the finger took place at points where the rapture of the skin layer occurred as a result of built-up shrinkage and syneresis stress that could not be relieved by creep relaxation. The accumulated stresses result in further penetration of non-solvent from the coagulation bath [37,75,76].

The penetration of non-solvent into the casted polymer solution to form the finger-like structure was hindered and minimized to a certain extent as the substrate was endowed with more intrusions and roughness. The casted membrane on a rougher surface showed minimal penetration of the fingers throughout the cross section of the membrane (i.e., PVDF-S150 with 9–20 µm, PVDF-S440 with 53 µm) compared to casting on a glass substrate, PVDF-G, with 7–83 µm. The porosity of the membrane is an important element that affects the transport of water vapor across the membrane. As can be seen from the cross section illustrated in Figure 6, all the membranes developed in this work, regardless of the substrate, demonstrated a porous, sponge-like structure. However, the nature of the substrate affected the amount and size of pores in the membrane matrix. The porosity enhanced as a result of casting on the rougher surface (compare Figure 6b vs. Figure 6l), which can be attributed to the role of the surface roughness during the phase-inversion process. A rough surface provides more anchor to the casted film in a way that minimizes potential shrinkage resulting from the solvent and non-solvent exchange and the formation of polymer-rich and polymer-lean regions. Thus, larger intrusions from the substrate allow more traction of the casted polymer solution against the shrinkage [77]. Conversely, smooth surfaces allow the casted polymer to contract and shrink, and ultimately resulted in smaller pores and a dense matrix. The variation of thickness within a membrane indicates the amount of and extent of penetration of the abrasive particles on the casted solution. Since S150 sandpaper is endowed with larger particles, the doped solution covered the particles from the top and between the particles. Thus, PVDF-S150 exhibited a variation in thickness from 72 to 164 µm. Such deep penetration would have a significant influence on the porosity of the membrane. This coincides well with the porosity measurements. These findings indicate the key role of substrate nature in determining the membrane characteristics, including the contact angle, porosity, pore size range and surface roughness.

**Figure 7:**
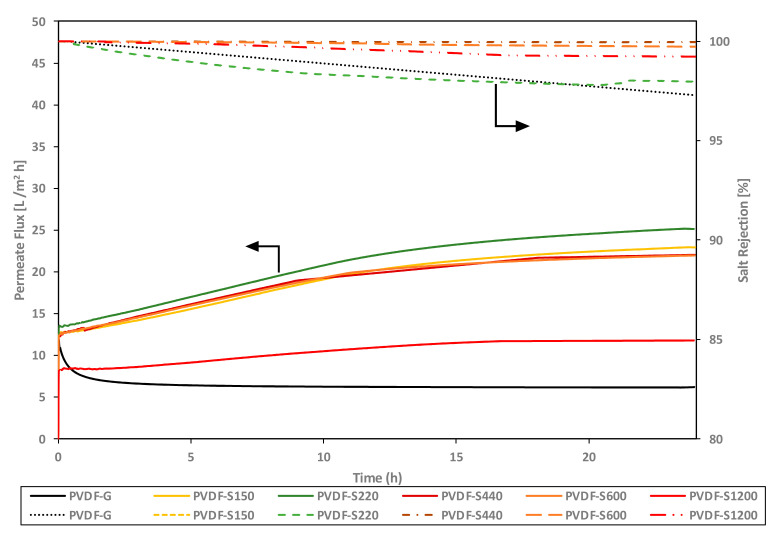
MD performance of PVDF-G and PVDF-SX series membranes for the desalination of highly saline water (feed concentration = 70,000 ppm; feed temperature = 70 °C) using air-gap membrane distillation (AGMD).

In membrane distillation, to evaluate the performance of the membrane there are two critical values that need to be considered: salt rejection and flux. The salt rejection indicates the ability of the membrane to prevent the flow of liquid (membrane wetting) across the membrane. Membranes with high contact angles and optimum porosity will result in higher LEP values that will eventually help the membranes to prevent the liquid phase to penetrate through the membrane and only allow water vapor to pass through. Flux is the indication of the amount of water vapor that can pass across the membrane per unit of area and time. Membranes with larger pores and open channels can help significantly in increasing the flux of the membrane. Balancing the increase in flux while maintaining high rejection requires careful optimization [78]. Figure 7 presents the salt rejection performance over 24 h for membranes fabricated using both types of substrates (i.e., sandpapers and glass). All the membranes showed similar performance in the first 3 h. Although most of the membrane salt rejection decreased over time to a minimum salt rejection of 97% after a course of 24 h, the PVDF-S400 and PVDF-S600 membranes showed stable 100% salt rejection over 24 h. Notably, casting on the glass, as is typically practiced in most of the literature related to membrane distillation, resulted in a membrane with deteriorated salt rejection from 100% to 97% over 24 h. Such stability over 24 h can be attributed to the nature of the membrane surface and how the nature increases the resistance of membrane wettability.

While casting on glass resulted in a membrane with a low flux value (7 L/m^2^·h), casting on sandpaper substrates resulted in a significant increase in the membrane flux. Casting on 1200 sandpaper (slightly rougher than the glass surface) resulted in more than a 75% increase in the flux. This percentage was significantly increased as a result of casting on sandpaper with a lower grit size. For instance, casting on sandpaper with grit size S600–S150 enhanced the flux by at least 210%. The increase in the membrane flux indicates a higher percentage of porosity, larger pore size, or both. Casting on sandpaper prevented membrane shrinkage during formation (see Table 2); therefore, the membranes would possess more and/or larger channels for the water vapor to transport across the membrane. The lower shrinkage witnessed when casted on sandpaper can be attributed to the intrusions (abrasive particles) from the sandpaper surface, which can help in holding the membrane against shrinkage and ultimately supporting the membrane in maintaining its porosity. Selecting the best membranes with the greatest potential requires consideration of both salt rejection and water flux. For example, while casting on PVDF-S150 resulted in a higher value for flux, the membrane had the worst salt rejection performance. Although PVDF-S150 possessed a contact angle similar to PVDF-S400 (casted on sandpaper with 400 grit size), the larger abrasive particles in S150 sandpaper held the membrane better during the phase inversion and accordingly resulted in slightly larger pores (and one of the lowest LEPs reported in this study). Notably, as can be seen in Figure 7, there was no direct correlation between the sandpaper grit size and the trend in the MD performance. Interestingly, the developed membranes in this study outperformed commercial membranes and other previously reported membranes (as can be seen in Table 3). These findings can help in delineating the key role of substrate nature in designing highly efficient membranes for membrane distillation.

## 4. Conclusions

In this study, a series of PVDF membranes with tunable wettability resistance and porosity were fabricated via a single and scalable approach. A wide range of sandpaper grit sizes were utilized as substrates for casting the polymer solution. Although all the membranes were fabricated using the same polymer, solvent, sandpaper materials and fabrication conditions, variations in the roughness of the substrate could enhance the hydrophobicity and tune the porosity of the membranes. Casting on sandpapers resulted in PVDF-SX hydrophobic membranes with water contact angles up to 105° (compared with PVDF-G, which had a CA of around 84°). The typically faced shrinkage of a polymeric membrane made via phase inversion was significantly reduced (and in some cases eliminated) as the sandpaper intrusions (abrasive particles) in the casted dope solution helped to prevent membrane contraction. Thus, the developed membranes on sandpapers possessed higher porosity and larger pores. Notably, the textured surface varied in terms of contact angle. Thus, increasing the pore size does not necessarily result in decreasing the LEP, as the LEP value is influenced by both max pore size and contact angle. PVDF-SX series membranes demonstrated stable salt rejection (up to 100%) and at least a 210% increase in the permeate flux over 24 h. Hence, the findings in this study can pave the way for the facile and scalable fabrication of highly efficient microporous polymeric PVDF membranes for wide industrial applications.

## Figures and Tables

**Table 1:** Testing conditions used for evaluating the MD performance using the AGMD system.

Parameters		Values
Effective membrane area		7.3 × 10^−4^ m^2^
Coolant	Temperature	20 ± 0.3 °C
	Flow rate	2 L/min
Feed	Salinity	70 g/L
	Temperature	70 ± 0.8 °C
	Flow rate	0.8 L/min
	Width of the air gap	≈0.002 m

**Table 2:** Results of porometric analysis, liquid entry pressure (LEP), thickness and shrinkage of membrane dimension compared to the casted dope solution dimension of PVDF-G and PVDF-S series membranes.

Samples	Maximum Pore Size (µm)	Mean Pore Size (µm)	Minimum Pore Size (µm)	Porosity (%)	Thickness (µm)	LEP (bar)	Shrinkage (%)
PVDF-G	0.21	0.18	0.16	73.2	100.4	0.8	8
PVDF-S150	0.44	0.39	0.35	81	145.5	0.5	0
PVDF-S220	0.39	0.35	0.30	78.1	138.2	0.6	0
PVDF-S400	0.38	0.34	0.30	74.8	117.5	1.0	0
PVDF-S600	0.37	0.32	0.26	75.2	124.2	0.7	0
PVDF-S1200	0.35	0.33	0.30	74	110.0	0.5	5.8

**Table 3:** Comparison of the PVDF-SX reported in this work with other membranes tested in the MD system.

Membrane	PTFE Commercial	PVDF Commercial	PVDF Hierarchically Textured Superhydrophobic Membrane Fabricated via Nanocasting	PVDF Superhydrophobic Membrane with Hierarchical 3D Microtexture	PVDF-S400
	[79]	[80]	[26]	[81]	(This work)
Air Gap (mm)	Air gap (NA)	Air gap (2 mm)	Direct contact	Direct contact	Air gap (2 mm)
Feed TDS (wt% NaCl)	5.8	6	3.5	3.5	7
Feed Temp. (°C)	50	80	60	60	70
Feed Flow rate (L/min)	1.5	0.35	1.08	0.6	08
Coolant Temp. (°C)	10	20	20	20	20
Coolant Flow rate (L/min)	8.5	1.2 m/s	1.08	0.5	2
Flux (L/m^2^·h)	10.8 (Duration: NA)	9 (Duration: NA)	14 (Duration: 24 h)	24 (Duration: only 3 h)	20 (Duration: after 24 h)

## Data Availability

Not applicable.
